# Activation of NF-κB in Basolateral Amygdala Is Required for Memory Reconsolidation in Auditory Fear Conditioning

**DOI:** 10.1371/journal.pone.0043973

**Published:** 2012-09-05

**Authors:** Jijian Si, Jianli Yang, Lifen Xue, Chenhao Yang, Yixiao Luo, Haishui Shi, Lin Lu

**Affiliations:** 1 Tianjin Medical University, Tianjin, China; 2 Tianjin Mental Health Center, Tianjin, China; 3 National Institute on Drug Dependence, Peking University, Beijing, China; University of Queensland, Australia

## Abstract

Posttraumatic stress disorder (PTSD) is characterized by acute and chronic changes in the stress response, manifested as conditioned fear memory. Previously formed memories that are susceptible to disruption immediately after retrieval undergo a protein synthesis-dependent process to become persistent, termed reconsolidation, a process that is regulated by many distinct molecular mechanisms that control gene expression. Increasing evidence supports the participation of the transcription factor NF-κB in the different phases of memory. Here, we demonstrate that inhibition of NF-κB in the basolateral amygdala (BLA), but not central nucleus of the amygdala, after memory reactivation impairs the retention of amygdala-dependent auditory fear conditioning (AFC). We used two independent pharmacological strategies to disrupt the reconsolidation of AFC. Bilateral intra-BLA infusion of sulfasalazine, an inhibitor of IκB kinase that activates NF-κB, and bilateral intra-BLA infusion of SN50, a direct inhibitor of the NF-κB DNA-binding complex, immediately after retrieval disrupted the reconsolidation of AFC. We also found that systemic pretreatment with sodium butyrate, a histone deacetylase inhibitor that enhances histone acetylation, in the amygdala rescued the disruption of reconsolidation induced by NF-κB inhibition in the BLA. These findings indicate that NF-κB activity in the BLA is required for memory reconsolidation in AFC, suggesting that NF-κB might be a potential pharmacotherapy target for posttraumatic stress disorder.

## Introduction

Posttraumatic stress disorder (PTSD) is characterized by acute and chronic changes in the stress response, manifested as a conditioned fear memory [1], [2]. The persistence of learned fear responses to danger-related cues may result in excessive fear and anxiety. Recent research has focused on the reconsolidation phase of fear memories to ameliorate PTSD [3], [4]. Many studies have indicated that when a stabilized memory is reactivated, it again becomes sensitized to disruption and must undergo new protein synthesis to regain stabilization, which is termed reconsolidation [5], [6], [7]. Protein translation mechanisms have been widely implicated as a necessary component of memory reconsolidation [5], but the gene transcription mechanisms are not well known. The reconsolidation of contextual conditioned fear (CCF) memory is subject to disruption through inhibition of protein synthesis or inhibition of signaling cascades, such as the extracellular signal-regulated kinase-mitogen-activated protein kinase (ERK/MAPK) pathway [8], [9]. Previous studies indicated that nuclear factor-κB (NF-κB) is required for synaptic plasticity, animal behavior, and long-term memory formation [6], [10], [11]. Our recent findings showed that NF-κB inhibition impairs the reconsolidation of morphine reward memory in rats [12].

The NF-κB family of dimeric transcription factors consists of five mammalian members: RelA (p65), RelB, c-Rel, NF-κB1 (p105/p50), and NF-κB2 (p100/p52) [13]. NF-κB is localized mainly to the cytoplasm in an inactive form bound to an inhibitory κB protein (IκB). Activation of NF-κB facilitates translocation to the nucleus and binding to the promoter region of target genes by recognizing the κB consensus sequence within DNA [14]. In addition to NF-κB having a well-characterized function in the immune system, other studies have indicated that it is also involved in the central nervous system [4], [15].

Previous evidence indicated that NF-κB is activated during memory consolidation in the context signal memory paradigm in the crab *Chasmagnathus*, in which memory formation is highly correlated with NF-κB activation [16]. An electrophoretic mobility shift assay of nuclear extracts from fear-trained rats showed a selective increase in NF-κB DNA binding activity in the amygdala, and intra-amygdala infusion of κB decoy DNA prior to training impaired fear consolidation [17]. Accumulating studies have confirmed the involvement of NF-κB specifically in the regulation of neuronal plasticity and memory formation. p65- and p50-containing NF-κB complexes have been shown to be involved in synaptic activation and memory formation [18]. NF-κB binding activity in the hippocampus in mice is strongly induced by spatial learning in the Morris water maze, but p65^−/−^ mice showed spatial learning impairments [19], [20]. Additionally, NF-κB is involved in the reconsolidation of contextual fear conditioning. NF-κB is specifically activated by brief exposure to the training context, and this reactivation is required for memory reconsolidation [16], [21].

The amygdala is the central structure involved in both fear conditioning and extinction [22]. Previous studies showed that AFC was associated with cyclic adenosine monophosphate (cAMP) response element binding protein (CREB) activation and cAMP-response element-mediated transcription selectively in the amygdala [23]. However, in contextual fear conditioning, CREB significantly increases in areas CA1 and CA3 of the hippocampus [24], [25]. The basolateral amygdala (BLA) receives information about conditioned and unconditioned stimuli, in contrast to the central nucleus of the amygdala (CeA), which receives information processed in the BLA [26]. Neuronal projections from the CeA to the hypothalamus and brainstem then mediate behavioral, autonomic, and emotional responses to stressful and fearful stimuli [27]. Although the role of NF-κB in memory has been extensively studied, unknown is whether NF-κB in the amygdala is required for the reconsolidation of AFC. Therefore, we used an AFC memory to explore the potential role of NF-κB in the amygdala in memory reconsolidation and its underlying mechanisms.

## Results

### Inhibition of IKK Activity with Sulfasalazine in the Basolateral Amygdala after Retrieval Impairs the Reconsolidation of Long-term Auditory Fear Conditioning Memory

**Figure 1:**
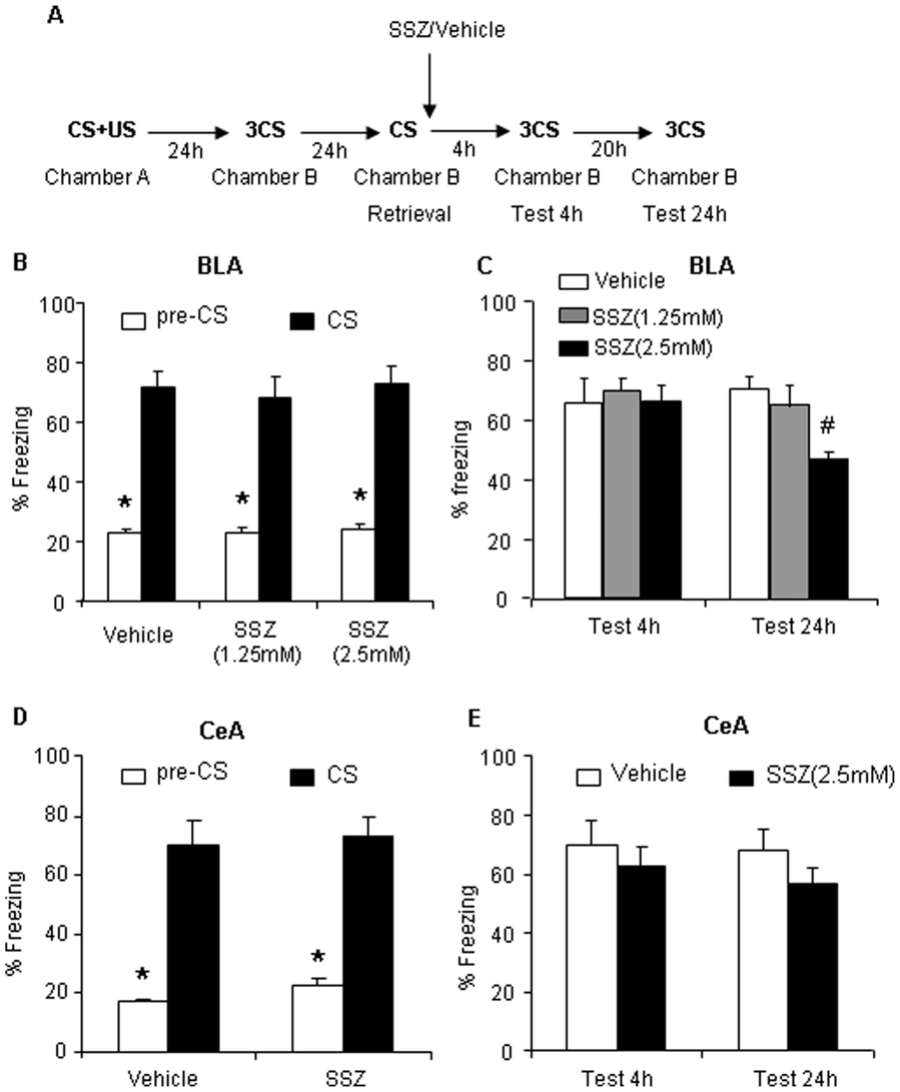
Inhibition of IKK activity in the BLA after retrieval impairs the reconsolidation. (A) Outline of the experimental procedure. (B) Freezing time in response to the CS after training was comparable across groups that received bilateral intra-BLA infusion of SSZ or vehicle (vehicle, *n* = 11; 1.25 mM SSZ, *n* = 9; 2.5 mM SSZ, *n* = 10). (C) Freezing behavior after bilateral intra-BLA infusion of SSZ or vehicle in the 4 h test was comparable across groups, but the 24 h SSZ (2.5 mM/side) infusion group exhibited significant amnesia. (D) Freezing time in response to the CS after training was comparable across groups that received an intra-CeA infusion of SSZ or vehicle (vehicle, *n* = 8; 2.5 mM SSZ, *n* = 10). (E) Freezing behavior test after intra-CeA infusion of SSZ or vehicle in the 4 h and 24 h tests (*p*>0.05). The data are expressed as mean ± SEM. *p<0.01, compared with pre-C; ^#^ *p*<0.01, compared with vehicle group (**two-wa**y ANOVA followed by *post hoc* test).

To assess the effect of inhibiting NF-κB signaling on memory recall, we infused sulfasalazine (SSZ), a direct pharmacological inhibitor of IKK [10], [21], [28], into the BLA and CeA to identify the specific regions in which NF-κB signaling plays a critical role in the reconsolidation of long-term AFC memories. Fig. 1A shows a schematic illustration of the behavioral procedure that depicts the time-points at which SSZ was administered and when freezing behavior was tested. Two different doses of SSZ (1.25 and 2.5 mM/side) were infused into the BLA, and a high dose of SSZ (2.5 mM/side) was infused into the CeA immediately after memory retrieval, and their effects were assessed 4 and 24 h after retrieval. In the 4 h test, no significant differences in freezing behavior were observed between the groups of animals used for subsequent vehicle and SSZ treatment (Fig. 1C). In the 24 h test, the lower dose of SSZ (1.25 mM/side) infused into the BLA immediately after retrieval showed a trend toward a decrease, but no significant difference in freezing behavior was found compared with vehicle-treated animals (p>0.05). However, animals infused with the higher dose of SSZ (2.5 mM/side) into the BLA (Fig. 1C), but not into the CeA (Fig. 1E), showed significantly less freezing over tests (F2,29 = 9.424, p<0.01). No significant differences in freezing behavior were observed between groups in the 4 h test, but reduced freezing behavior was observed in the 24 h test in the animals that received SSZ administration, indicating that SSZ administration after retrieval interfered with the reconsolidation process but did not enhance extinction memory. These results indicate that the NF-κB pathway in the BLA, but not in the CeA, participates in the retrieval-induced reconsolidation of long-term AFC memory but not short-term memory.

### NF-κB Signaling Activity after Memory Recall is Specific to Auditory Reexposure

**Figure 2:**
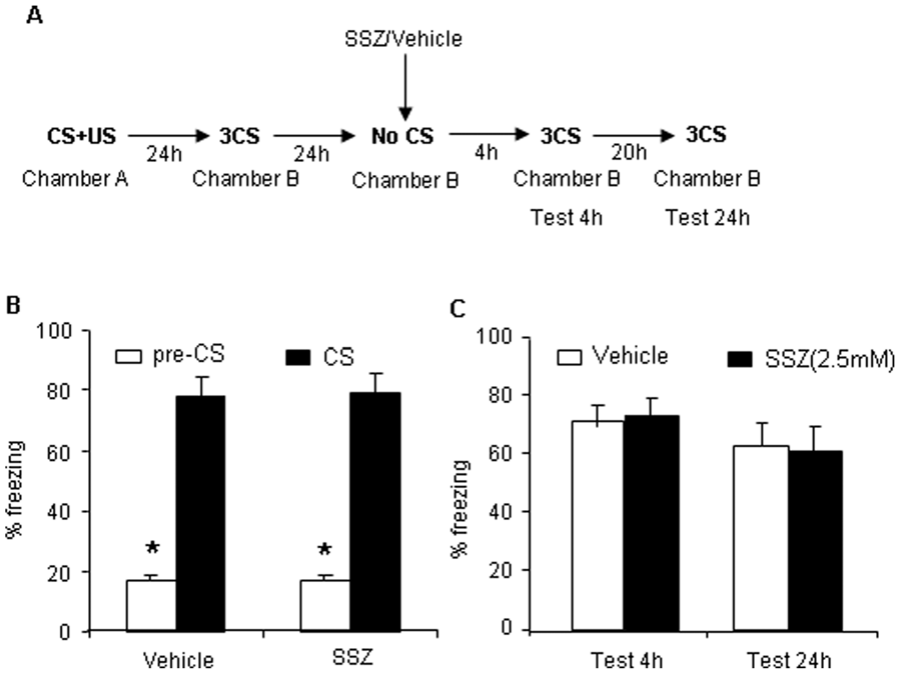
The effect of SSZ is specific to reexposure to auditory conditioning. (A) Outline of the experimental procedure. (B) Freezing time in response to the CS after training was comparable across groups that received bilateral intra-BLA infusion of SSZ or vehicle (vehicle, *n* = 10; SSZ, *n* = 9). (C) No significant difference in freezing time was observed after bilateral intra-BLA infusion of SSZ or vehicle in the 4 h and 24 h tests between groups (*p*>0.05). The data are expressed as mean ± SEM. *p<0.01, compared with pre-CS.

To evaluated whether NF-κB activation in the BLA is specific to auditory retrieval induced by reexposure to a shock-paired tone in another context (context B), the following experiment was conducted. Twenty-four hours after post-conditioning fear test, vehicle or SSZ (2.5 mM/side) was infused into the BLA, similarly to Experiment 1, with the exception that the retrieval trial was not conducted (Fig. 2A). No significant difference was found in either the vehicle or SSZ groups in the 4 h and 24 h tests (Fig. 2B). These results indicate that NF-κB activation occurred only when the animals were reexposed to the same cue previously used during training.

### Sulfasalazine Injected into the Basolateral Amygdala before Retrieval has no Effect on the Expression of Conditioned Fear Memory

**Figure 3:**
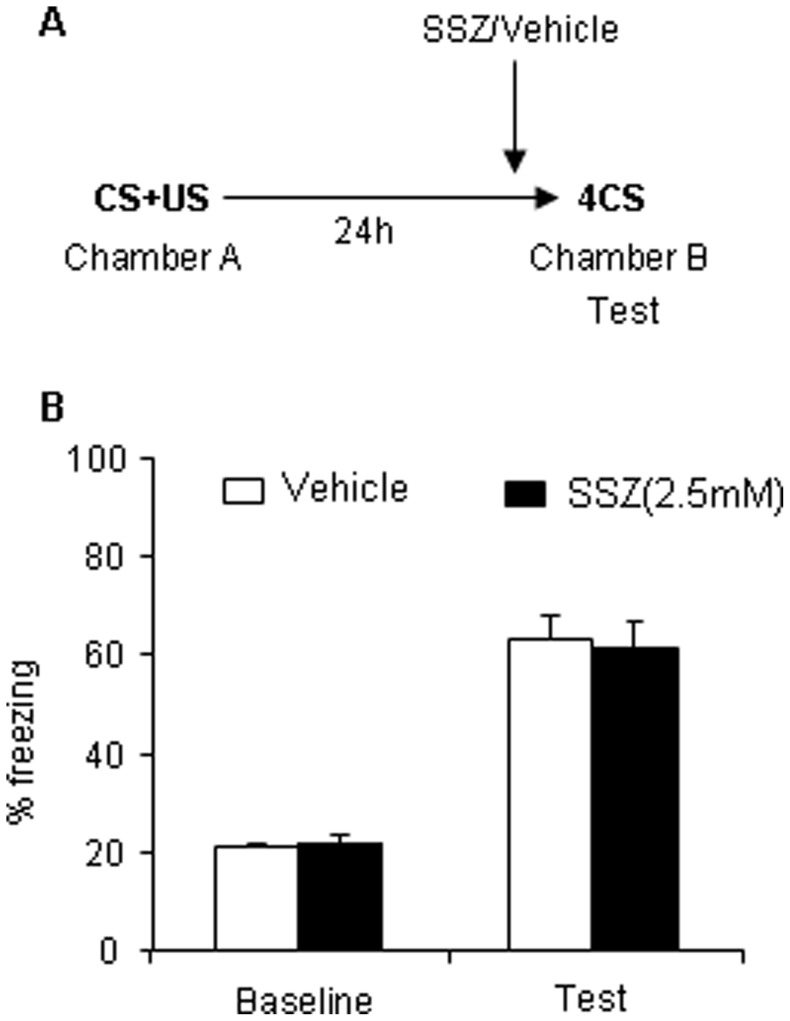
Intra-BLA infusion of SSZ before retrieval has no effect on the expression of AFC memory. (A) Outline of the experimental procedure. (B) Freezing behavior test on the next day after training. No significant difference was found between the two groups (vehicle, *n* = 10; SSZ, *n* = 10). The data are expressed as mean ± SEM. *p>0.05, compared with vehicle group.

This experiment assessed the effect of bilateral intra-BLA infusion of SSZ on the expression of conditioned fear memory. Twenty-four hours after post-conditioning fear test in context A, the animals received bilateral intra-BLA infusion of SSZ or vehicle 20 min before the freezing test in context B. Fig. 3A shows the behavior procedure. No significant difference was observed between the two groups in the test. Bilateral intra-BLA infusion of SSZ 20 min before the test had no effect on the expression of auditory fear memory.

### Direct Inhibition of the NF-κB Transcriptional Complex with SN50 after Retrieval Impairs the Reconsolidation of Long-term Auditory Fear Conditioning Memory

**Figure 4:**
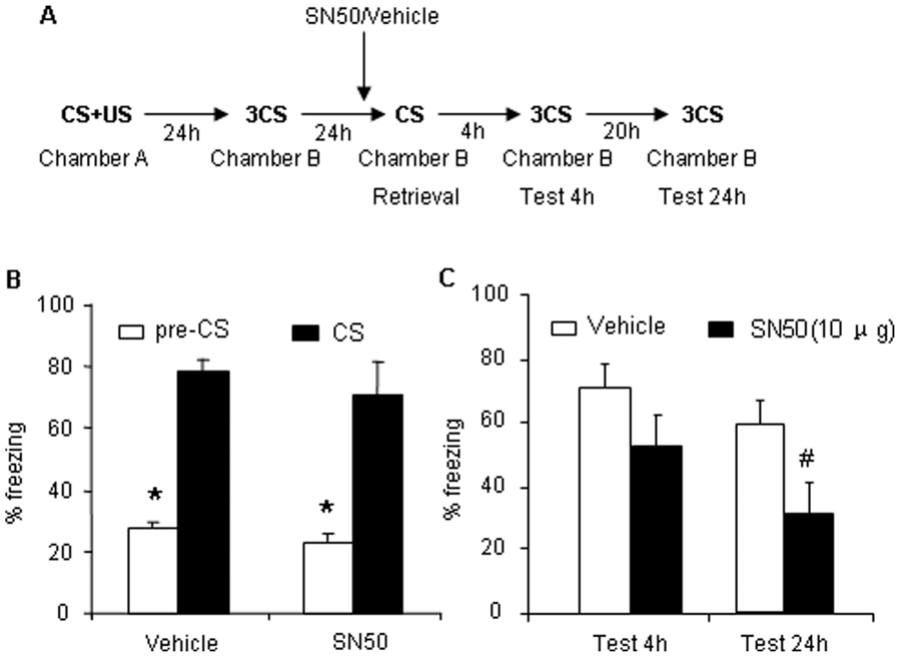
Direct inhibition of NF-κB with SN50 impairs the reconsolidation of long-term AFC memory. (A) Outline of the experimental procedure. (B) Freezing time in response to the CS after training was comparable across groups that received bilateral intra-BLA infusion of SN50 or vehicle (vehicle, *n* = 8; SN50, *n* = 9). *p<0.01, compared with vehicle group. (C) Freezing behavior test after bilateral intra-BLA infusion of SN50 or vehicle in the 4 h and 24 h tests. In the 24 h test, freezing behavior in rats that received bilateral intra-BLA infusion of SN50 was significantly lower than in rats treated with vehicle. The data are expressed as mean ± SEM. *p<0.01, compared with pre-CS; ^#^ *p*<0.01, compared with vehicle group (two-way ANOVA followed by *post hoc* test).

This experiment assessed whether direct inhibition of the NF-κB DNA-binding complex by SN50 (10 µg/side) 2 hours prior to retrieval impairs AFC memory [21]. The experimental procedure was similar to Experiment 1, with the exception that infusion of SSZ immediate after retrieval trial was substituted by infusion of SN50 2 hours prior to retrieval trial (Fig. 4A). In the 4 h test, no significant differences in freezing behavior were observed between these two groups, indicating that bilateral intra-BLA infusion of SN50 or vehicle 2 h prior to reexposure did not impair short-term AFC memory (Fig. 4B). In the 24 h test, the group that was treated with bilateral intra-BLA infusion of SN50 exhibited a significant decrease in freezing behavior over tests (*F_1,16_* = 6.803, *p*<0.05, Fig. 4B). These results indicate that bilateral intra-BLA infusion of SN50 immediately after retrieval impaired the reconsolidation of long-term AFC memory in the 24 h test but had no effects on short-term memory in the 4 h test.

### The Effect of SN50 is Specific to Reexposure to Auditory Fear Conditioning

**Figure 5:**
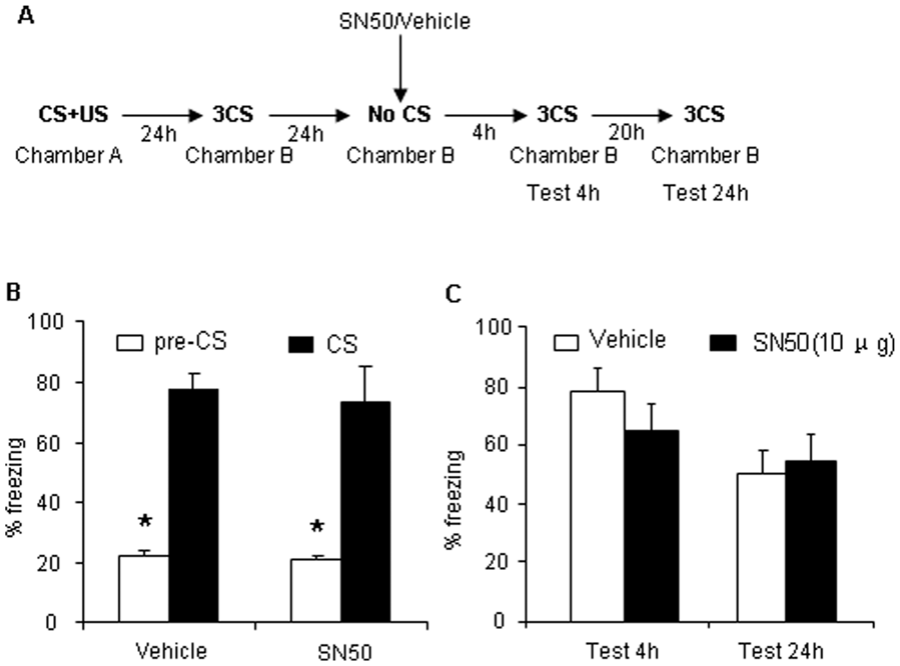
The effect of SN50 is specific to reexposure to auditory conditioning. (A) Outline of the experimental procedure. (B) Freezing time in response to the CS after training was comparable across groups that received bilateral intra-BLA infusion of SN50 or vehicle (vehicle, *n* = 9; SN50, *n* = 9). (C) No significant difference in freezing time was observed after bilateral intra-BLA infusion of SN50 or vehicle in the 4 h and 24 h tests between groups (*p*>0.05). The data are expressed as mean ± SEM. *p<0.01, compared with pre-CS (two-way ANOVA followed by *post hoc* test).

This experiment evaluated whether the impairment induced by bilateral intra-BLA infusion of SN50 is specific to reexposure to AFC. Twenty-four hours after post-conditioning fear test, the animals received bilateral intra-BLA infusion of vehicle or SN50 at the same time as in Experiment 4 but without a retrieval trial (Fig. 5A). No significant difference was observed between the two groups that received bilateral intra-BLA infusion of vehicle (0.9% saline, 1 µl/side) or SN50 (10 µg/side), respectively, in the short-term and long-term memory tests (Fig. 5B). These results indicate that NF-κB activation occurred only when the animals were reexposed to the same cue previously used during training.

### Sodium Butyrate Rescued Memory Reconsolidation in the Presence of Sulfasalazine-induced IKK Inhibition

**Figure 6:**
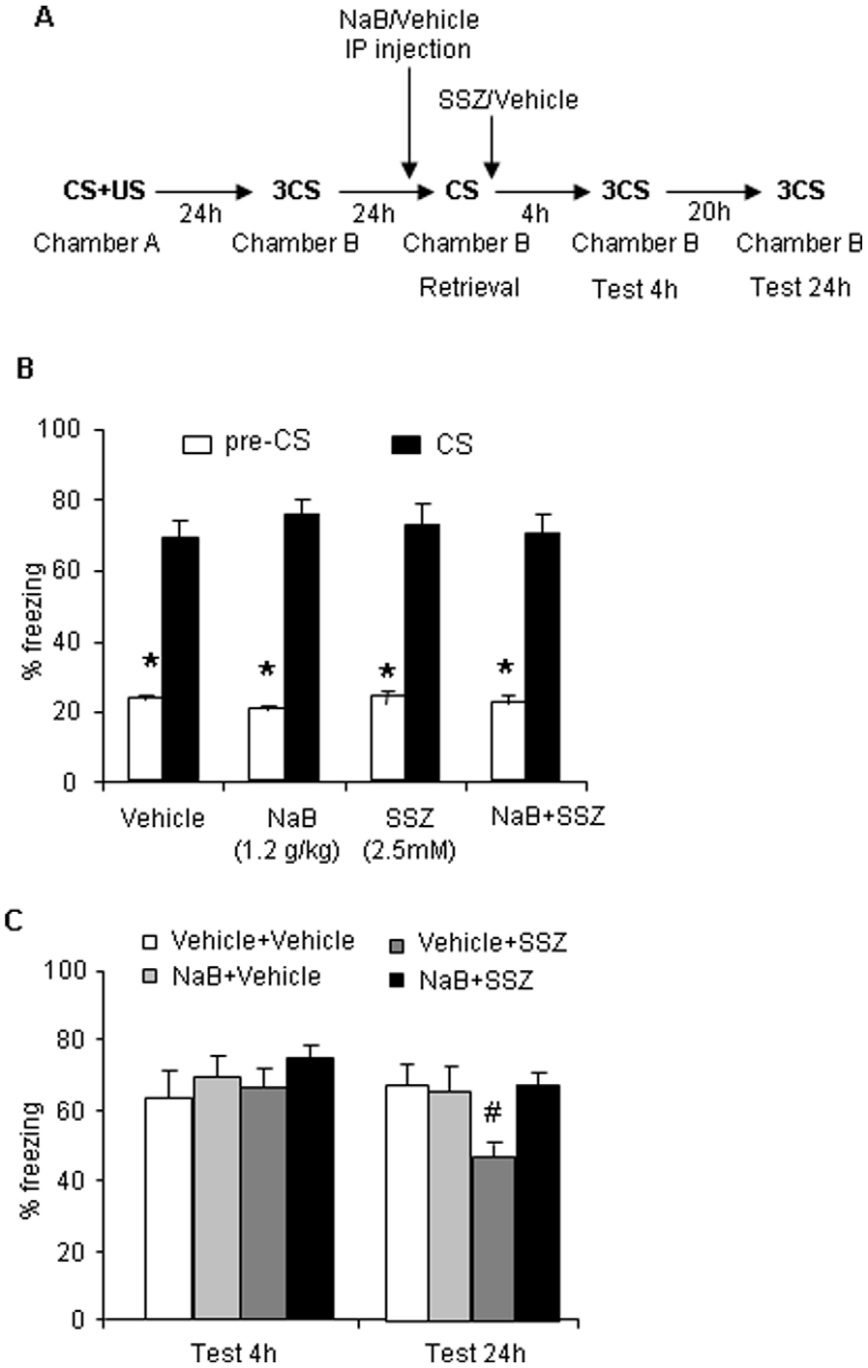
Sodium butyrate (NaB) could rescue memory reconsolidation in the presence of IKK inhibition with SSZ. (A) Outline of the experimental procedure. (B) Freezing time in response to the CS after training was comparable across groups and was specific to the CS (vehicle + vehicle, *n* = 10; vehicle + SSZ, *n* = 9; NaB + vehicle, *n* = 9; NaB + SSZ, *n* = 10). *p<0.01, compared with pre-CS. (C) No significant difference in freezing behavior was observed between groups in the 4 h test. Freezing behavior in rats that received the NaB + SSZ treatment in the 24 h test was significantly lower compared with rats that received the vehicle + SSZ treatment (*F* = 4.702, *p*<0.05). The data are expressed as mean ± SEM. ^#^ *p*<0.01, compared with the group that received the vehicle + SSZ treatment (two-way ANOVA followed by *post hoc* test).

This experiment evaluated whether augmenting chromatin remodeling through activation of histone acetylation reverses the impairment of reconsolidation induced by inhibition of NF-κB in the BLA immediately after retrieval. We used the HDAC inhibitor sodium butyrate (NaB) to enhance histone acetylation and determined whether this manipulation could rescue the impaired long-term AFC memory induced by IKK inhibition in the BLA immediately after retrieval. The animals were trained as described previously. Twenty-four hours after post-conditioning fear test, the animals received an injection of vehicle or NaB (1.2 g/kg, i.p.) 1 h prior to reexposure to the training cue in context B (Fig. 6A). One hour prior to reexposure to the training cue in context B, the rats were separated into two groups that received vehicle (1 ml/kg) or NaB (1.2 g/kg, i.p.). Each group then received bilateral intra-BLA infusion of vehicle (0.5 µl/side) or SSZ (2.5 mM/side) immediately after retrieval. The freezing behavior test was performed 4 h and 24 h later. In the 4 h test, no significant difference in freezing behavior was observed among the four groups that received the different treatments. These results indicate that NaB had no effect on short-term memory. As expected, the conditioned freezing behavior in rats that received the vehicle + SSZ treatment was significantly disrupted compared with the rats that received the vehicle + vehicle treatment, and the impairment of AFC was rescued by pretreatment with NaB (*F_3,37_* = 5.741, *p*<0.05; Fig. 6B). These results indicate that enhancing histone acetylation was sufficient to ameliorate the effect of inhibiting NF-κB activity on AFC memory reconsolidation.

## Discussion

The present study identified the participation of the IKK/NF-κB signal pathway in the reconsolidation of AFC in rats. We found that inhibition of the NF-κB signaling cascade at the level of IKK protein kinase or the NF-κB DNA binding complex in the BLA, but not CeA, blocked the reconsolidation of long-term AFC memory and that inhibition of the NF-κB signaling cascade at the level of IKK protein kinase had no effect on the expression of AFC. Additionally, the impairment of the reconsolidation of long-term AFC induced by inhibition of the NF-κB signaling cascade in the BLA was rescued by pretreatment with a histone deacetylation inhibitor.

Previous studies have shown that the amygdala is a critical part of emotional processing, supported by a large body of evidence from fear conditioning investigations. The amygdala is a central structure that plays pivotal roles in fear memory formation and reconsolidation [5], [22], [29]. Studies of the molecular mechanisms of long-term fear memory have implicated the NF-κB signaling pathway in the processes of both consolidation and reconsolidation [18], [21], [30], [31]. Compared with contextual fear conditioning, which is a hippocampus-dependent contextual fear memory, AFC is considered a classic model of fear memory that involves the amygdala [17], [31]. The role of the NF-κB signaling pathway in the hippocampus-dependent contextual fear memory reconsolidation has been well documented [21]. However, little is known about the role of NF-κB cascades in the BLA in memory reconsolidation. Therefore, we focused on identifying the role of these molecular markers in the amygdala in reconsolidation of auditory fear memories.

Activation of the NF-κB signaling pathway requires upregulation of the IKK complex in hippocampus-dependent contextual fear memory [16], [32]. In the present study, we found that inhibition of NF-κB signaling significantly reduced the expression of long-term AFC memory but not short-term memory, indicating that activation of the NF-κB signaling pathway in the BLA is involved in the reconsolidation of long-term AFC memory.

In the present study, SSZ and SN50 were used to inhibit the activity of NF-κB. SN50 is a direct inhibitor of the NF-κB DNA-binding complex, and SSZ is a direct pharmacological inhibitor of IKK, both of which inhibit the activity of NF-κB [28]. In despite of no direct molecular evidence that NF-κB in the BLA was activated after retrieval trial in the current study, previous studies have demonstrated that NF-κB signaling pathway activity in hippocampus is nessesary for reconsolidation of contextual fear memory that is hippocampus-dependent memory [21]. The experiments used a 4 h test trial to demonstrate that SSZ and SN50 administration after retrieval had no effects on short-term memory and only interfered with reconsolidation. Although the animals underwent a 4 h test during which three CSs were delivered, freezing behavior in the vehicle group was not different from the post-training test, 4 h test, or 24 h test. Thus, the three CSs presentations during the 4 h test did not affect the 24 h test; therefore, we did not perform an experiment in which the animals were tested for freezing behavior only in the 24 h test with no 4 h test. Additionally, protein synthesis in the BLA is required for reconsolidation of auditory fear memory [5], [22], [29]. Therefore, in our study we directly investigated the role of NF-κB in the BLA in reconsolidation of auditory fear memory by pharmacological inhibition of NF-κB activity. We found that bilateral intra-BLA infusion of SSZ immediately after retrieval or bilateral intra-BLA infusion of SN50 2 h prior to retrieval disrupted the reconsolidation of AFC and that inhibition of NF-κB activity in the BLA had no effects on stable memories, suggesting that NF-κB activity is upregulated by retrieval and plays a critical role in the reconsolidation of auditory fear memory. The results are consistent with a previous study of hippocampus-dependent contextual fear conditioning [21]. NF-κB activation regulates synaptic plasticity and long-term memory by controlling gene expression after transfer to the nucleus and binding to the κB consensus sequence [33]. Therefore, inhibition of NF-κB during the reconsolidation phase disrupted long-term AFC.

Previous studies have shown that histone acetyltransferases (HATs) were activated in the hippocampus during memory reconsolidation [12], [34]. NaB-induced inhibition of histone deacetylase not only enhanced histone acetylation but also enhanced NF-κB acetylation, which increased NF-κB DNA binding complex activity [21], [31]. Numerous studies suggest that chromatin remodeling allows robust and lasting changes in gene expression, particularly in the nervous system [35], [36]. Several mechanisms have been described for the NF-κB signaling pathway that have been shown to be involved in the regulation of gene expression through the modification of histone phosphorylation and acetylation in concert with histone deacetylase (HDAC) in non-neuronal cells [10], [37], [38].

In the present study, NaB, an inhibitor of HDAC that enhances histone acetylation, was used to explore whether the augmentation of chromatin remodeling induced by increasing histone acetylation can rescue the impairment of long-term AFC induced by inhibition of NF-κB activity in the BLA during reconsolidation phase. The results showed that the impairment of long-term AFC induced by inhibition of NF-κB activity in the BLA during the reconsolidation phase was reversed by systemic pretreatment with NaB, suggesting that freezing behavior deficits induced by IKK inhibition in the BLA after memory recall was rescued by enhancing histone acetylation induced by pretreatment with NaB. Although we found that HDAC inhibition rescues memory deficits caused by NF-κB blockade, the straightforward molecular mechanism underlying this effect needs to be further studied.

According to a previous study, increased DNA binding activity of NF-κB induced by HDAC inhibitor enhanced fear-potentiated startle, which indicated that the interaction between histone acetylation and NF-kB pathway may be functional [21], [31]. However, no enhancement in freezing behavior was found in the animals infused with NaB alone, which is contrary to earlier published results [34], [35], [39]. This effect may be ascribed to the training protocol which may be too strong to observe the enhancement of freezing time induced by systemic delivery of NaB. According to our present findings, we may conclude that NF-κB activity regulated by histone deacetylase mechanisms in the BLA was involved in the reconsolidation of auditory fear memory.

In conclusion, our results suggest that the NF-κB signaling pathway in the BLA is required for the reconsolidation of AFC through the regulation of gene transcription mechanisms. Additionally, fear amnesia induced by blocking NF-κB activity in the BLA during the reconsolidation phase could be rescued by pretreatment with an HDAC inhibitor. Activation of the NF-κB pathway is required for the reconsolidation of AFC memory, and histone deacetylation may be an intra-nuclear molecular switch that culminates in the termination of the NF-κB transcriptional response in long-term memory. Our findings may provide a novel target to help people release traumatic memories and a novel pharmacological intervention for the treatment of PTSD.

## Materials and Methods

### Animals

One hundred eighty Sprague-Dawley rats (weighing 240–260 g upon arrival) were obtained from the Peking University Experimental Animal Center. The animals were housed under a 12 h/12 h light/dark cycle and allowed ad libitum access to rodent chow and water. The room temperature was maintained at 21–23°C and 45–50% relative humidity. The rats were handled daily during the first week after arrival. All experimental procedures in the current study were performed in accordance with the National Institutes of Health Guide for the Care and Use of Laboratory Animals and were approved by Biomedical Ethics Committee of Peking University of animal use and protection.

### Surgery

During surgery, each rat was implanted with a 23-gauge stainless steel cannula (Plastics One, Roanoke, VA, USA) into the BLA (coordinates: 2.9 mm posterior to bregma, 5.0 mm lateral to midline, and 8.5 mm ventral to skull surface) or CeA (coordinates: 2.9 mm posterior to bregma, 4.2 mm lateral to midline, and 7.8 mm ventral to skull surface according to our previous study [40]. The rats were given at least 5 days to recover before the experimental procedures began.

### Drugs and Injection Procedures

**Figure 7:**
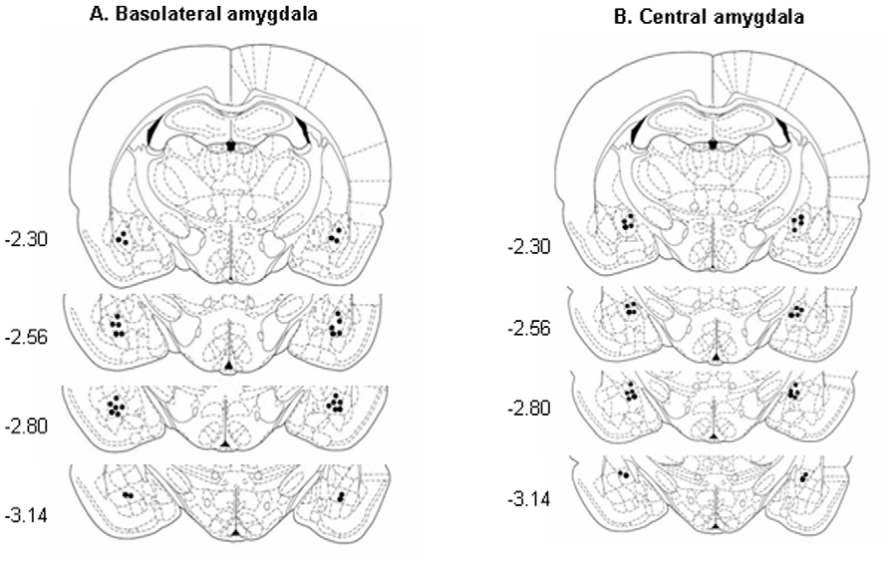
Schematic illustration of the injection sites in the BLA and CeA. The black dots indicate the locations of the cannula tips [41].

For the experiments that investigated the effect of inhibiting the NF-κB signaling pathway on long-term memory after recall, the animals received an infusion of either vehicle or SSZ into the BLA (1.25 and 2.5 mM/side; Sigma, St. Louis, MO) or CeA (2.5 mM/side). Vehicle was saline solution with 10 mM HEPES (pH 7.6) plus 20% dimethyl sulfoxide (DMSO; final pH 7.6) and SSZ was freshly dissolved in vehicle. All infusions were performed immediately following the retrieval session in chamber B. For the experiments that investigated the effect of inhibiting the NF-κB DNA-binding complex on long-term memory after recall, the SN50 active peptide (10 µg/side) or saline was infused into the BLA. SN50 infusions were performed 2 h prior to the retrieval session in chamber B. SN50 was dissolved in saline. SSZ or SN50 were bilaterally infused into BLA or CeA with Hamilton syringes connected to 30 gauge injectors (PlasticsOne). The infusion volume was 0.5 µl, and the drug was injected bilaterally over 1 min; the injection needle was kept in place for an additional 1 min to allow for drug diffusion [40] **.** For the experiments that investigated the ability of NaB to prevent the disruption of long-term memory produced by central SSZ administration, the animals were injected with NaB (1.2 g/kg, i.p., dissolved in saline) 1 h prior to the retrieval session. After all of the behavioral tests, the rats were anesthetized with sodium pentobarbital (100 mg/kg, i.p.) and transcardially perfused with paraformaldehyde (4%, pH 7.4). Cannula placements were assessed using Nissl staining with a section thickness of 40 µm under light microscopy. The rats with misplaced cannulae were excluded [40]. The locations of the cannula tips are shown in Fig. 7 [41].

### Apparatus

Fear conditioning and tone testing were conducted in different chambers. For conditioning, the rats were placed in a Plexiglas rodent conditioning chamber (context A) with a grid floor and a white light. For tone testing, the rats were placed in a different Plexiglas chamber (context B) that was brightly lit with three house lights and had a black floor that had been washed with peppermint soap. A micro-video camera was mounted at the top of the chamber so that the rats could be videotaped during testing.

### Fear Conditioning

The animals were handled for 5 days after surgery. On the day of the experiments, they were transported to the laboratory at least 2 h prior to fear conditioning. The animals were placed into training chamber A and allowed to explore it for 10 min. Fear conditioning was then conducted with three 20 s, 5 kHz, 80 dB tones (conditioned stimulus [CS]), each co-terminating with a 1 s, 1 mA footshock (unconditioned stimulus [US]; [3]. The interval between each CS was an average of 120 s. After conditioning, each rat was returned to its home cage. For the behavioral experiments, freezing behavior was assessed 4 and 24 h after the retrieval of fear conditioning. Retrieval was performed in context B with a single tone. Freezing behavior of the rats during the training or test phase was observed by micro-video camera (absence of all but respiratory movement was defined as freezing behavior). Behavior was manually scored by a rater blind to the experimental conditions. The total amount of CS-induced freezing, expressed as a percentage of total CS, was used as a measure of fear.

### Data Analysis

The data are expressed as the mean ± standard error of the mean (SEM). Data in all experiments were analyzed with two-way analysis of variance (ANOVA) followed by the Tukey *post hoc* test. Values of *p*<0.05 were considered statistically significant.
